# Association between physician-staffed helicopter versus ground emergency medical services and mortality for pediatric trauma patients: A retrospective nationwide cohort study

**DOI:** 10.1371/journal.pone.0237192

**Published:** 2020-08-12

**Authors:** Yuki Enomoto, Asuka Tsuchiya, Yusuke Tsutsumi, Koji Ishigami, Junpei Osone, Masahito Togo, Susumu Yasuda, Yoshiaki Inoue

**Affiliations:** 1 Department of Emergency and Critical Care Medicine, Faculty of Medicine, University of Tsukuba, Ibaraki, Japan; 2 Department of Emergency and Critical Care Medicine, National Hospital Organization Mito Medical Center, Ibaraki, Japan; 3 Department of Clinical Epidemiology and Health Economics, School of Public Health, The University of Tokyo, Tokyo, Japan; Lundquist Institute at Harbor-UCLA Medical Center, UNITED STATES

## Abstract

**Background:**

Helicopter emergency medical services’ (HEMS) effectiveness for pediatric trauma patients remains unclear. We aimed to examine the relation between HEMS and reduced mortality in pediatric trauma patients.

**Methods:**

This retrospective cohort study utilized data from the Japan Trauma Data Bank, a national multicenter clinical trauma database. Participants were aged <18 years, admitted between 2004 and 2015, and transported from the scene to the hospital by HEMS or ground emergency medical services (GEMS). We used a standardized mortality ratio (SMR) weight method, and fitted a marginal structural model to adjust for measured confounders. The SMR weight was calculated using the estimation of the propensity scores. A logistic regression model was used with the baseline independent variables to estimate the propensity score.

**Results:**

Overall, 5,947 patients were identified in our study: 453 were transported by HEMS and 5,494 by GEMS. The mean injury severity score in the HEMS group was significantly higher than that in the GEMS group17.0 (Standard deviation = 11.0) vs 12.2 (Standard deviation = 9.2), *p* < .001. In-hospital mortality was higher in the HEMS group than that in the GEMS group in the unadjusted analysis (3.8% vs 1.3%, respectively; *p* < .001). After adjusting for covariates, HEMS transport was not associated with reduced hospital mortality. (odds ratio = 0.82, 95% confidence interval = 0.42–1.58).

**Conclusions:**

HEMS was not associated with reduced mortality among pediatric trauma patients compared with GEMS in this nationwide study. Further investigation is necessary to determine who clearly benefits from HEMS as compared to GEMS.

## Introduction

Helicopter emergency medical services (HEMS) are an integral part of the emergency medical transport system. HEMS have been used to transport emergency patients from the scene to the hospital in many developed countries [1–11]. A systematic review in 2015 concluded that, compared to ground emergency medical services (GEMS), HEMS were not related to a reduction in adult patient mortality rates [1]. However, following the report’s publication, some studies noted that, compared to GEMS, HEMS were more highly associated with reducing mortality rates in adult trauma patients [2–5]. In the 2015 systematic review, subgroup analyses suggested that HEMS might be associated with reducing mortality in studies that adjusted appropriately for confounders. Proper statistics might delineate and clarify any association.

However, the relationship between HEMS transfer and pediatric trauma patient mortality is still poorly understood. In some studies, HEMS were reported to be associated with reducing mortality among pediatric trauma patients [6–8]; however, other reports showed that they were not related to improved patient outcomes [10]. Notably, all studies addressing pediatric trauma patients were conducted in the United States; the impact of HEMS (relative to GEMS) has not been reported in other countries.

HEMS have also been reported as being overused [8, 12], sometimes being called for non-severe pediatric patients; therefore, it is not clear whether their effectiveness is only associated with patients with severe trauma. Moreover, HEMS cost more than GEMS [13, 14], and staff require in-depth training concerning aviation and medical services [15]. Consequently, it is essential to investigate the association of HEMS with reduction in the pediatric trauma patient mortality. This study aimed to determine the relation between HEMS and mortality in pediatric trauma patients transported from the emergency scene to the hospital.

## Materials and methods

### Study design and setting

This was a multicenter retrospective cohort study. We obtained permission to use data from the Japan Trauma Data Bank (JTDB), established in 2003 by the Japanese Association for the Surgery of Trauma (Trauma Registry Committee), and the Japanese Association for Acute Medicine (Committee for Clinical Care Evaluation). The JTDB was established to collect and investigate data on trauma patients in Japan, and to share suggestions about trauma management with clinicians. In 2017, 272 hospitals voluntarily submitted data to the JTDB [16], and 93% of the data were collected from tertiary-level emergency hospitals [2]. About 74% of the Japanese tertiary-level emergency hospitals from every prefecture have contributed to the database [16]. The registration criterion of the JTDB is typically an abbreviated injury scale (AIS) score ≥ 3; however, patients who have less severe injuries can be registered. To maintain the quality, those submitting an AIS were considered to have had AIS code training. Data cleansing was performed by Japan Trauma Care and Research.

The Japanese HEMS were developed in 2001, sparked by the Hanshin-Awaji Earthquake in 1995. Today, 53 HEMS have been implemented all over Japan, and they are now deployed in about 90% of all Japanese prefectures. The Japanese HEMS are staffed by one to two physicians, who are board-certified in fields such as emergency care, surgery, and anesthesiology, and approximately 28,000 patients are transported by HEMS annually [17].

Data concerning pediatric cases that have been transported by HEMS require elucidation. Hospitals specifically for the treatment of severely injured children do not exist in Japan, and children’s hospitals usually do not provide care for severe trauma patients. However, some emergency centers that typically treat adult critical patients also serve severely injured pediatric trauma patients, although pediatricians may not be available for full-time duty in some of those centers [18].

In addition, the Japanese GEMS differ from those in Western countries. The Japanese GEMS can only administer drugs in limited cases: intravenous (IV) epinephrine for cardiopulmonary arrest, intramuscular epinephrine for anaphylaxis (when prescribed epinephrine by a doctor), and IV glucose for hypoglycemia. Therefore, GEMS in Japan rarely perform medical interventions, especially among pediatric patients [18].

### Participants

The data of JTDB from 2004 to 2015 were utilized. Inclusion criteria for the study comprised participants aged < 18 years, and who were transported from the accident scene to a hospital by HEMS or GEMS. Only children arriving at a hospital from 8 a.m. to 6 p.m. were included because HEMS only operate during the daytime.

The following cases were excluded in line with previous studies: those experiencing pediatric cardiopulmonary arrest (because of its lethality), or pediatric burn victims (because of the difference from other traumas) [6, 10]. In addition, we excluded cases that did not list the cause of the trauma, the victim’s vital signs at the scene, or the outcome. Patients with missing length of hospital stay were excluded only when ICU days were analyzed as an outcome.

### Variables

The data collected included age, sex, type of incident, first vital signs at the scene (heart rate, systolic blood pressure, respiratory rate, and consciousness level), severity of injured body part (AIS), injury severity score (ISS), and whether or not emergency surgery was performed. Information on time was also collected: time from the call until reaching hospital, time taken to reach the scene and then the hospital, time from arrival at the hospital to blood transfusion and then to surgery. Additionally, information regarding the unit the patient was transferred to from the emergency room (ER) was also collected. Issues such as hospital mortality, length of hospital stays, and discharge to home (vs transferred to another hospital unit) were also analyzed. The primary outcome was in-hospital mortality; the secondary outcome was the length of hospital stay.

Patients were categorized into six age groups: infants (aged < 1 year), toddlers (aged 1–2 years), preschoolers (aged 3–5 years), school-aged (aged 6–9 years), preadolescents (aged 10–12 years), and adolescents (aged 13–17 years). Vital signs were classified into three groups according to the patient’s age: normal, above normal, and below normal [19]. For details concerning vital signs, please see S1 Table.

The level of consciousness was evaluated using the Japan Coma Scale (JCS) score, which is commonly used in Japan, including for children [20, 21] Patients were categorized into four groups based on JCS score: 0 (Grade 0, alert); 1–3 (Grade 1, distracted); 10–30 (Grade 2, somnolence); and 100–300 (Grade 3, coma) [22]. Emergency surgery was defined as surgery that was performed within three hours of hospital arrival [9], emergency blood transfusion was defined as blood transfusion performed within two hours of hospital arrival, and a severe injury was defined as an AIS score ≥ 3.

The following cases in JTDB were considered as missing data: transport time from the scene to the hospital > 120 minutes (because there is appropriate access to hospitals in Japan, transport time should not take more than two hours), and time from hospital arrival to surgery and/or emergency blood transfusion > 48 hours (because taking > 48 hours indicates that it is not an emergency).

### Statistical analyses

Continuous variables were presented as means and standard deviations. Ordinal variables were presented as median and interquartile ranges. Categorical variables were presented as numbers and percentages. Comparisons were performed with t-tests for continuous variables, Wilcoxon rank-sum test for ordinal variables, and chi-squared tests for categorical variables. We also showed the characteristics of the participating children, and those who died in the hospital. Since transport mode was not randomly selected, we used a standardized mortality ratio weight method, and fitted a marginal structural model to adjust for measured confounders [23, 24]. Regarding the weighting of these clinical parameters, it may be noted that the distribution of the confounders and the number of patients in the GEMS group (weighted population) are equivalent to those in the HEMS group. Therefore, we can directly compare GEMS and HEMS on an equal basis. The statistical technical details were based on marginal structural model theory, and the inverse-probability-of-treatment weighting method described in other technical literature [25]. A propensity score was used as a conditional probability of HEMS, considering all confounders. A logistic regression model was applied with the baseline independent variables (age, sex, ISS, anatomical location of the severe injury, and categorized vital signs at the scene) to estimate the propensity score. Covariates were carefully selected based on the assumption that they were not directly affected by the intervention. For the standardized mortality ratio weight, treated patients (HEMS) were assigned a weight of one, while the weight for control patients (GEMS) was calculated as follows: estimated propensity score divided by one minus the estimated propensity score. The ratio reweights the GEMS to be comparable to the HEMS population. Last, we calculated the weighted logistic and the linear regression analyses to estimate the odds ratio and mean difference for the mortality and the length of hospital stay. As with the HEMS, we only analyzed daytime transport. The decision to use GEMS or HEMS was influenced not only by patients’ conditions but also by demographics or weather conditions. Consequently, a marginal structural model was suitable for our study objectives.

In this analysis, complete cases were used for all comparisons. All tests of significance were two-tailed, and *p* < .05 was considered significant. Variables were analyzed with Stata version 14 (Stata-Corp, College Station, Texas, USA).

### Subgroup analyses

HEMS were reported to be effective only for severe trauma patients (ISS > 15) [8, 12]; therefore, subgroup analyses were performed on patients with ISS > 15 and ≤ 15 to confirm the relevance of severity and HEMS effectiveness. In addition, a close proximity to hospital may diminish the benefits of HEMS because of the shortened transport time [6]. A subgroup analysis was thus performed based on transport time to clarify its effects: > 15 and ≤ 15 minutes.

### Sensitivity analysis

In view of the long period of data used, changes in the management of childhood trauma due to the changing times may have influenced the result. Therefore, we added the year of injury to the propensity score, and performed a sensitivity analysis.

### Ethics statement

This study was approved by the institutional review board of the National Hospital Organization Mito Medical Center (2017–19), which waived the requirement for informed patient consent because of the anonymous nature of the data.

## Results

**Fig 1:**
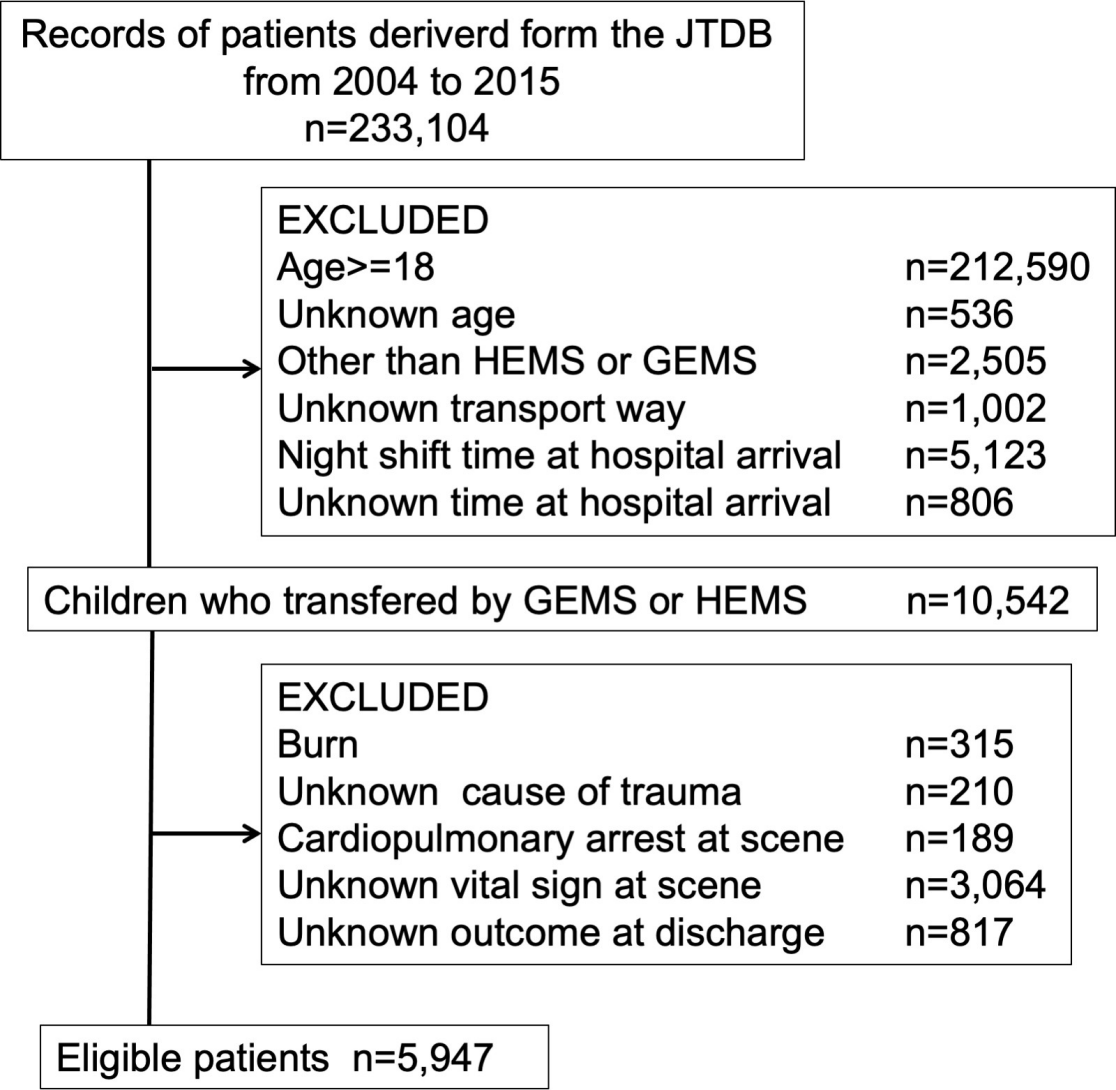
Participant selection of patients from the Japan Trauma Data Bank 2004–2015. The number of patients excluded in the HEMS and the GEMS groups was 38 and 277, respectively, for burns, 50 and 160 for unknown causes of trauma, 22 and 167 for cardiopulmonary arrest at scene, 408 and 2656 for unknown vital signs at scene, and 52 and 765 for unknown outcomes at discharge. JTDB, Japan Trauma Data Bank; HEMS, helicopter emergency medical services; GEMS, ground emergency medical service.

Overall, 5,947 patients were identified in our study: 453 transported by HEMS and 5,494 by GEMS (Fig 1). Patients’ characteristics are shown in Table 1. Significantly, more patients were transported by HEMS than GEMS in cases involving traffic accidents and worse vital signs at the scene. Patients in the HEMS group took longer than those in the GEMS group to be transported from the scene to a hospital. Median ISS in the HEMS group was significantly higher than in the GEMS group. Concerning post-hospital data, more children were admitted to the intensive care unit in the HEMS group; however, the number of patients who needed emergency surgery was similar in both the groups. Nearly half of the emergency surgeries were for bone fixation. Fewer patients were discharged to home directly from the emergency critical care department in the HEMS group than in the GEMS group.

**Table 1:** Characteristics of children transported by emergency medical services.

Characteristics	HEMS n = 453	GEMS n = 5494	p-value
Age, mean (SD)	10.5 (4.7)	10.7 (4.5)	0.38
**Age category, n (%)**			0.10
Infants (0–1)	5 (1.1)	35 (0.6)	
Toddlers (1–2)	24 (5.3)	185 (3.4)	
Preschoolers (3–5)	36 (7.9)	464 (8.4)	
School-aged (6–9)	134 (29.6)	1631 (29.7)	
Preadolescents (10–12)	66 (14.6)	995 (18.1)	
Adolescents (13–17)	188 (41.5)	2184 (39.8)	
Sex (female), n (%)	125 (27.6)	1514 (27.6)	0.99
**Incident Type, n (%)**			<0.001
** Blunt**	445 (98.2)	5422 (98.7)	
Motor Vehicle	48 (10.6)	246 (4.5)	
Motorcycle	49 (10.8)	462 (8.4)	
Bicycle	110 (24.3)	1469 (26.7)	
Pedestrian	117 (25.8)	1151 (21.0)	
Fall	49 (10.8)	656 (11.9)	
Tumble	25 (5.5)	446 (8.1)	
Sport	24 (5.3)	523 (9.5)	
Other Blunt Injury	23 (5.1)	469 (8.5)	
Penetrating	8 (1.8)	72 (1.3)	
**Prehospital SBP, n (%)**			0.33
Normal	228 (50.3)	2944 (53.6)	
Hypotension	16 (3.5)	154 (2.8)	
Hypertension	209 (46.1)	2396 (43.6)	
**Prehospital HR, n (%)**			0.001
Normal	323 (71.3)	4323 (78.7)	
Bradycardia	9 (2.0)	76 (1.4)	
Tachycardia	121 (26.7)	1095 (19.9)	
**Prehospital RR, n (%)**			<0.001
Normal	213 (47.0)	3106 (56.5)	
Bradypnea	16 (3.5)	172 (3.1)	
Tachypnea	224 (49.4)	2216 (40.3)	
**JCS category, n (%)**			<0.001
Grade 0 (Alert)	130 (28.7)	2806 (51.1)	
Grade 1 (Distracted)	126 (27.8)	1500 (27.3)	
Grade 2 (Somnolence)	67 (14.8)	555 (10.1)	
Grade 3 (Coma)	93 (20.5)	467 (8.5)	
missing	37 (8.2)	166 (3.0)	
**Head & Neck Injury, n (%)**			<0.001
AIS < 3	242 (53.4)	3616 (65.8)	
AIS ≥ 3	211 (46.6)	1878 (34.2)	
unknown	0 (0)	0 (0)	
**Chest Injury, n (%)**			<0.001
AIS < 3	325 (71.7)	4674 (85.1)	
AIS ≥ 3	128 (28.3)	819 (14.9)	
unknown	0 (0)	1 (<0.1)	
**Abdominal Injury, n (%)**			0.15
AIS < 3	419 (92.5)	5170 (94.1)	
AIS ≥ 3	34 (7.5)	320 (5.8)	
unknown	0 (0)	4 (0.1)	
**Extremities Injury, n (%)**			0.29
AIS < 3	354 (78.1)	4173 (76.0)	
AIS ≥ 3	99 (21.9)	1321 (24.0)	
unknown	0 (0)	0 (0)	
ISS, median (interquartile ranges)	16 (9–25)	9(5–16)	<0.001
Emergency Surgery (Hospital arrival to surgery ≤ 3 hours), n (%)	54 (11.9)	564 (10.3)	0.27
Emergency Blood Transfusion (Hospital arrival to blood transfusion ≤ 2 hours), n (%)	15 (3.3)	84 (1.5)	0.004
**Disposition after ED, n (%)**			<0.001
ICU Admission	353 (77.9)	3347 (60.9)	
General Ward	88 (19.4)	1966 (35.8)	
death at ED	1 (0.2)	5 (0.1)	
Others	11 (2.4)	120 (2.2)	
Missing	0 (0)	56 (1.0)	
In-Hospital Mortality, n (%)	17 (3.8)	73 (1.3)	<0.001
Length of Hospital Stay, mean (SD)	20.0 (28.7)	17.0 (34.9)	0.08
Discharge to Home, n (%)	305 (67.3)	4625 (84.2)	<0.001
Time from call to a hospital, mean minutes (SD)	54.5 (19.5)	36.9 (15.0)	<0.001
Time from a scene to a hospital, mean minutes (SD)	25.7 (18.0)	15.3 (11.7)	<0.001
Time from Hospital Arrival to Surgery, mean hour (SD)	1.9 (0.7)	1.9 (0.7)	0.81
Time from Hospital Arrival to Blood Transfusion, mean hour (SD)	1.1 (0.5)	1.1 (0.6)	0.94

Normal range of vital signs (upper limit of SBP, HR, and RR); 104 mmHg, 60–180 bpm, and 29–53 bpm in infants, respectively; 106 mmHg, 60–140 bpm, and 21–37 bpm in toddlers, respectively; 112 mmHg, 60–120 bpm, and 19–28 bpm in preschool-aged children, respectively; 115 mmHg, 60–118 bpm, and 17–25 bpm in school-aged children, respectively; 120 mmHg, 60–118 bpm, and 17–25 bpm in preadolescents, respectively; and 131 mmHg, 60–100 bpm, and 11–20 bpm in adolescents, respectively. Hypotension was defined as 70 + (2*Age) mmHg for children aged < 10 years and < 90 mmHg for children aged ≥ 10 years.

HEMS, helicopter emergency medical service; GEMS, ground emergency medical service; SD, standard deviation; SBP, systolic blood pressure; HR, heart rate; RR, respiratory rate; AIS, abbreviated injury scale; ISS, injury severity score; JSC, Japan Coma Scale; ED, emergency department; BPM, beat per minute for heart rate and breath per minute for respiration rate.

The characteristics of children who died in the hospital are shown in S2 Table. More than 70% in both the groups had been in coma, and more than half the patients had severe head or neck trauma; several others had severe chest or abdominal trauma. The ISS scores in both the groups exceeded 30. Emergency surgery was conducted on less than half of the patients.

**Fig 2:**
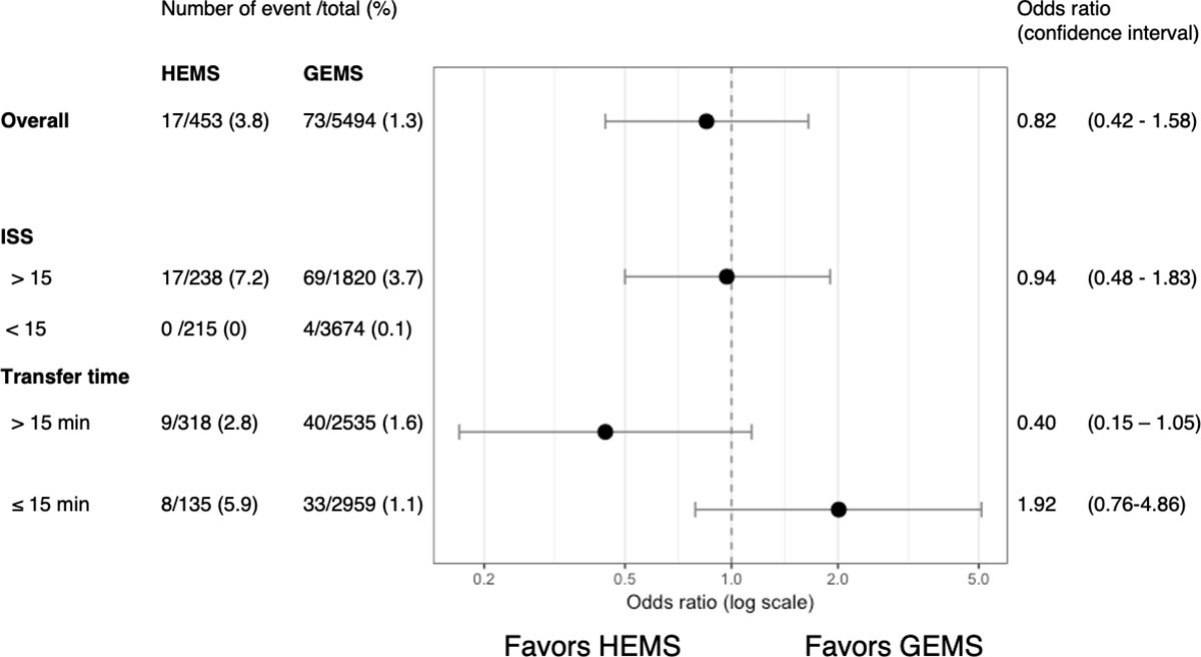
Odds ratios of in-hospital mortality between helicopter and ground emergency medical services. HEMS, helicopter emergency medical service; GEMS, ground emergency medical service; ISS, injury severity score; CI, confidence interval.

In-hospital mortality, using an unadjusted analysis in the HEMS group, was higher than that of the GEMS group (Table 1). When evaluating outcomes after adjusting for covariates, HEMS transport was not associated with hospital mortality (odds ratio (OR) = 0.82, 95% confidence interval (CI) = 0.42–1.58; Fig 2).

To determine the influence of severity of injury, and time from scene to hospital, we performed subgroup analyses. First, concerning severe trauma patients (ISS > 15), HEMS were not associated with hospital mortality (OR = 0.94; 95% CI = 0.48–1.83). In the ISS ≤ 15 subgroup, an OR could not be calculated because no patient died in the HEMS group. Second, concerning transport time, after adjusting for covariates, HEMS were not associated with hospital mortality: > 15 minutes (OR = 0.40, 95% CI = 0.15–1.06) and ≤ 15 minutes (OR = 1.92, 95% CI = 0.76–4.86).

Due to the missing length of hospital stay, 9 patients with HEMS and 85 patients with GEMS were excluded from the analysis for the length of hospital stay. There was no significant difference in the length of hospital stay between either of the two groups when unadjusted (Table 1). Further, there was no significant difference between HEMS and GEMS after adjusting for covariates (Table 2). In the subgroup analysis for severe trauma patients, HEMS were not associated with shortening the length of hospital stay. In the other subgroup analysis for transport time, neither group was associated with length of hospital stay.

**Table 2:** Mean difference in length of hospital stay between those receiving helicopter or ground emergency medical services.

Outcomes	Number of observation	Mean difference (95%CI)	
Overall*	5,853	-1.49	(-4.94 to 1.97)
**Subgroup analysis**			
Injury Severity Score			
> 15	2,031	-4.52	(-9.18 to 0.14)
≤ 15	3,822	2.19	(-2.50 to 6.88)
Time from scene to hospital			
>15 minutes	2,800	-1.01	(-4.54 to 2.51)
≤ 15 minutes	3053	-0.09	(-8.28 to 8.11)

*Due to missing the length of hospital stay, 94 patients were excluded from the analysis.

CI: Confidence Interval

In the sensitivity analysis, we added the year of injury to the analysis. There were no significant differences in in-hospital mortality (Odds ratio, 0.83; 95%CI, 0.43 to 1.61) and length of hospital stay (mean difference, -1.40; 95%CI, -4.86 to 2.07) between the HEMS and GEMS.

## Discussion

In this article, we applied a standardized mortality ratio weight method, and fitted a marginal structural model to study the effects of HEMS compared with GEMS for pediatric trauma patients in Japan. After adjusting for covariates, we did not find a significant difference between in-hospital mortality among patients who were transported via HEMS or GEMS.

Similar studies of pediatric trauma patients have demonstrated inconsistent results regarding the association of HEMS with reduced mortality compared to GEMS. The relation of HEMS to mortality reduction was not shown in a preceding study [10]; however, an association was revealed in larger studies [6–8] that examined reduced mortality in moderately to severely injured pediatric trauma patients.

There are some reasons why such an association for HEMS may not have been observed here. First, the injuries of our target patients might be too severe for them to be saved by early intervention. Head and neck injuries are common in pediatric trauma, and a serious head injury is one of the primary causes of death among pediatric trauma patients [26, 27]. Consistent with previous studies, the major causes of death for pediatric trauma in this study included coma, and severe head and neck injuries.

Further, more than half of the cases did not receive emergency surgery; however, the cases in the current study—in which the mean ISS values were 17.0 and 12.2 for HEMS and GEMS respectively—might have included more severe injuries than those in the previous studies, where the mean ISS ranged between 9.2 and 14 for HEMS, and between 6.7 and 9 for GEMS [7, 9, 28]. In our study, some children may have had injuries that were too severe to be saved by emergency surgery; therefore, emergency surgery was only performed in half of the cases.

Second, the Japanese health care system for pediatric trauma may still be underdeveloped. One of the chief components of the superiority of HEMS was that HEMS was reportedly a part of an organized trauma system [29].

Pediatric trauma centers may have been established in some countries (e.g., the United States, and EU countries), but we have not reached that level to establish such a health care system in Japan. We do not have a pediatric trauma center in Japan. While most children’s hospitals do not receive patients with severe trauma arriving directly from the scene of an accident, some emergency centers that do not have enough pediatricians accept severe pediatric trauma patients without reserve. In these conditions, the usefulness of HEMS might be limited.

Regarding transport time, our sample size of patients transported by HEMS might have been too small to perform stable analyses. Large studies are necessary to reveal the relationship between transport time and transport methods in mortality.

In addition, the results concerning our secondary outcome were like those of our primary outcome. The length of stay in hospital did not differ significantly according to transfer method. A prior study [9], which did not adjust for vital signs at the scene, described that patients treated with HEMS had longer lengths of stay (OR = 2.3, CI = 1.00–5.28). Another study, which partially adjusted for vital signs at the scene, described no significant difference arising from the transport mode [10]. Our results were consistent with the latter study, which included age-adjusted vital signs as covariates.

The major strength of this study was that we included categorized vital signs using age-adjusted standards as covariates, which are key predictors of patients’ deterioration [19, 30]. Only one separate study had used these [6], and another study had used these only regarding heart rate [10].

This study had several limitations. First, there was no significant difference in mortality between the GEMS and the HEMS groups, but the HEMS group had a lower mortality rate. Compared to previous reports indicating an association between HEMS and pediatric trauma mortality, our study had a small sample size. Therefore, a larger sample size may yield different results. Second, we did not collect information concerning the distance from the scene to a hospital, which may influence the decision to use HEMS. This variable might influence the longer pre-hospital total time in HEMS compared to GEMS. Third, we do not know whether the results of this study apply to countries other than Japan. In addition, patients without serious trauma are outside the scope of this database. Fourth, because of the database nature of the study, the granularity of the patient details was limited.

## Conclusions

In summary, in this nationwide study, compared to GEMS, HEMS were not associated with reduced hospital mortality among pediatric trauma patients. Future investigations are thus required to determine the effectiveness of HEMS, and to clearly identify who benefits from HEMS relative to GEMS.

## Supporting information

S1 Table Vital signs categories by age group. Normal range of vital signs (upper limit of SBP, HR, and RR); 104 mmHg, 60–180 bpm, and 29–53 bpm in infants, respectively; 106 mmHg, 60–140 bpm, and 21–37 bpm in toddlers, respectively; 112 mmHg, 60–120 bpm, and 19–28 bpm in preschool-aged children, respectively; 115 mmHg, 60–118 bpm, and 17–25 bpm in school-aged children, respectively; 120 mmHg, 60–118 bpm, and 17–25 bpm in preadolescents, respectively; and 131 mmHg, 60–100 bpm, and 11–20 bpm in adolescents, respectively. Hypotension was defined as 70 + (2*Age) mmHg for children aged < 10 years and < 90 mmHg for children aged ≥ 10 years. HEMS, helicopter emergency medical service; GEMS, ground emergency medical service; SD, standard deviation; SBP, systolic blood pressure; HR, heart rate; RR, respiratory rate; AIS, abbreviated injury scale; ISS, injury severity score; JSC, Japan Coma Scale; ED, emergency department. mmHg, millimeters of mercury; bpm, beat per minute for heart rate and breath per minute for respiration rate.
(DOCX)

S2 Table Characteristics of children who died at the hospital. Normal range of vital signs (upper limit of SBP, HR, and RR); 104 mmHg, 60–180 bpm, and 29–53 bpm in infants, respectively; 106 mmHg, 60–140 bpm, and 21–37 bpm in toddlers, respectively; 112 mmHg, 60–120 bpm, and 19–28 bpm in preschool-aged children, respectively; 115 mmHg, 60–118 bpm, and 17–25 bpm in school-aged children, respectively; 120 mmHg, 60–118 bpm, and 17–25 bpm in preadolescents, respectively; and 131 mmHg, 60–100 bpm, and 11–20 bpm in adolescents, respectively. Hypotension was defined as 70 + (2*Age) mmHg for children aged < 10 years and < 90 mmHg for children aged ≥ 10 years. HEMS, helicopter emergency medical service; GEMS, ground emergency medical service; SD, standard deviation; SBP, systolic blood pressure; HR, heart rate; RR, respiratory rate; AIS, abbreviated injury scale; ISS, injury severity score; JSC, Japan Coma Scale; ED, emergency department; mmHg, millimeters of mercury; bpm, beat per minute for heart rate and breath per minute for respiration rate.
(DOCX)
