# Supplementary material for: Association between physician-staffed helicopter versus ground emergency medical services and mortality for pediatric trauma patients: A retrospective nationwide cohort study
Source: PLoS One. 2020 Aug 12;15(8):e0237192. doi: 10.1371/journal.pone.0237192 (PMC7423096; doi:10.1371/journal.pone.0237192)
Supplement: S1 Table — Normal range of vital signs (upper limit of SBP, HR, and RR); 104 mmHg, 60–180 bpm, and 29–53 bpm in infants, respectively; 106 mmHg, 60–140 bpm, and 21–37 bpm in toddlers, respectively; 112 mmHg, 60–120 bpm, and 19–28 bpm in preschool-aged children, respectively; 115 mmHg, 60–118 bpm, and 17–25 bpm in school-aged children, respectively; 120 mmHg, 60–118 bpm, and 17–25 bpm in preadolescents, respectively; and 131 mmHg, 60–100 bpm, and 11–20 bpm in adolescents, respectively. Hypotension was defined as 70 + (2*Age) mmHg for children aged < 10 years and < 90 mmHg for children aged ≥ 10 years. HEMS, helicopter emergency medical service; GEMS, ground emergency medical service; SD, standard deviation; SBP, systolic blood pressure; HR, heart rate; RR, respiratory rate; AIS, abbreviated injury scale; ISS, injury severity score; JSC, Japan Coma Scale; ED, emergency department. mmHg, millimeters of mercury; bpm, beat per minute for heart rate and breath per minute for respiration rate. (DOCX) [file pone.0237192.s001.docx]

| Age Groups | Hypertension  (mmHg) | Hypotension  (mmHg) | Tachycardia (bpm) | Bradycardia (bpm) | Tachypnea (bpm) | Bradypnea (bpm) |
| --- | --- | --- | --- | --- | --- | --- |
| Infants  (<1 yo) | 104 | 70+(2*age) | 180 | 60 | 53 | 29 |
| Toddlers  (1-2 yo) | 106 | 70+(2*age) | 140 | 60 | 37 | 21 |
| Preschoolers  (3-5 yo) | 112 | 70+(2*age) | 120 | 60 | 28 | 19 |
| School-Aged  (6-9 yo) | 115 | 70+(2*age) | 118 | 60 | 25 | 17 |
| Preadolescents  (10-12 yo) | 120 | 90 | 118 | 60 | 25 | 17 |
| Adolescents  (13-17 yo) | 131 | 90 | 100 | 60 | 20 | 11 |

**S1 Table. Vital signs categories by age group.**
