# Supplementary material for: Association between physician-staffed helicopter versus ground emergency medical services and mortality for pediatric trauma patients: A retrospective nationwide cohort study
Source: PLoS One. 2020 Aug 12;15(8):e0237192. doi: 10.1371/journal.pone.0237192 (PMC7423096; doi:10.1371/journal.pone.0237192)
Supplement: S2 Table — Normal range of vital signs (upper limit of SBP, HR, and RR); 104 mmHg, 60–180 bpm, and 29–53 bpm in infants, respectively; 106 mmHg, 60–140 bpm, and 21–37 bpm in toddlers, respectively; 112 mmHg, 60–120 bpm, and 19–28 bpm in preschool-aged children, respectively; 115 mmHg, 60–118 bpm, and 17–25 bpm in school-aged children, respectively; 120 mmHg, 60–118 bpm, and 17–25 bpm in preadolescents, respectively; and 131 mmHg, 60–100 bpm, and 11–20 bpm in adolescents, respectively. Hypotension was defined as 70 + (2*Age) mmHg for children aged < 10 years and < 90 mmHg for children aged ≥ 10 years. HEMS, helicopter emergency medical service; GEMS, ground emergency medical service; SD, standard deviation; SBP, systolic blood pressure; HR, heart rate; RR, respiratory rate; AIS, abbreviated injury scale; ISS, injury severity score; JSC, Japan Coma Scale; ED, emergency department; mmHg, millimeters of mercury; bpm, beat per minute for heart rate and breath per minute for respiration rate. (DOCX) [file pone.0237192.s002.docx]

**S2 Table. Characteristics of children who died at the hospital**

| Characteristics | HEMS n=17 | GEMS n=73 | p-value |
| --- | --- | --- | --- |
| Age, mean (SD) | 12.7 (5.4) | 11.2 (4.6) | 0.25 |
| **Age category, n (%)** |  |  | 0.48 |
| Infants (0-1) | 0 ( 0) | 1 (1) |  |
| Toddlers (1-2) | 2 (12) | 4 (5) |  |
| Preschoolers (3-5) | 0 (0) | 1 (1) |  |
| School-aged (6-9) | 2 (12) | 24 (33) |  |
| Preadolescents (10-12) | 2 (12) | 10 (14) |  |
| Adolescents (13-17) | 11 (65) | 33 (45) |  |
| Sex (female), n (%) | 4 (24) | 25 (34) | 0.39 |
| **Incident Type, n (%)** |  |  | 0.18 |
| **Blunt** | 12 (100) | 72 (99) |  |
| Motor Vehicle | 3 (18) | 6 (8) |  |
| Motorcycle | 3 (18) | 6 (8) |  |
| Bicycle | 5 (29) | 13 (18) |  |
| Pedestrian | 1 (6) | 18 (25) |  |
| Fall | 1 (6) | 16 (22) |  |
| Tumble | 2 (12) | 2 (3) |  |
| Sport | 2 (12) | 7 (10) |  |
| Other Blunt Injury | 0 (0) | 4 (5) |  |
| Penetrating | 0 (0) | 1 (1) |  |
| **Prehospital SBP, n (%)** |  |  | 0.17 |
| Normal | 8 (47) | 29 (40) |  |
| Hypotension | 0 (0) | 13 (18) |  |
| Hypertension | 9 (53) | 31 (42) |  |
| **Prehospital HR, n (%)** |  |  | 0.63 |
| Normal | 10 (59) | 44 (60) |  |
| Bradycardia | 2 (12) | 4 (5) |  |
| Tachycardia | 5 (29) | 25 (34) |  |
| **Prehospital RR, n (%)** |  |  | 0.87 |
| Normal | 5 (29) | 26 (36) |  |
| Bradypnea | 3 (18) | 13 (18) |  |
| Tachypnea | 9 (53) | 34 (47) |  |
| **JCS category, n (%)** |  |  | 0.47 |
| Grade 0 (Alert) | 2 (12) | 4 (5) |  |
| Grade 1 (Distracted) | 1 (6) | 5 (7) |  |
| Grade 2 (Somnolence) | 0 (0) | 7 (10) |  |
| Grade 3 (Coma) | 12 (71) | 54 (74) |  |
| missing | 2 (12) | 3 (4) |  |
| **Head & Neck Injury, n (%)** |  |  | 0.11 |
| AIS < 3 | 6 (35) | 13 (18) |  |
| AIS ≥ 3 | 11 (65) | 60 (82) |  |
| unknown | 0 (0) | 0 (0) |  |
| **Chest Injury, n (%)** |  |  | 0.58 |
| AIS < 3 | 9 (53) | 44 (60) |  |
| AIS ≥ 3 | 8 (47) | 29 (40) |  |
| unknown | 0 (0) | 0 (0) |  |
| **Abdominal Injury, n (%)** |  |  | 0.15 |
| AIS < 3 | 16 (94) | 64 (88) |  |
| AIS ≥ 3 | 1 (6) | 9 (12) |  |
| unknown | 0 (0) | 0 (0) |  |
| **Extremities Injury, n (%)** |  |  | 0.29 |
| AIS < 3 | 15 (88) | 56 (77) |  |
| AIS ≥ 3 | 2 (12) | 17 (23) |  |
| unknown | 0 (0) | 0 (0) |  |
| ISS, median (interquartile ranges) | 38 (25-45) | 26 (25-39.5) | 0.24 |
| Emergency Surgery  (Hospital arrival to surgery ≤ 3 hours), n (%) | 8 (47) | 35 (48) | 0.95 |
| Emergency Blood Transfusion  (Hospital arrival to blood transfusion ≤ 2 hours), n (%) | 4 (24) | 23 (32) | 0.52 |
| Time from call to a hospital,  mean minutes (SD) | 57.6 (19.3) | 35.2 (13.9) | <0.001 |
| Time from a scene to a hospital,  mean minutes (SD) | 22.3 (18.6) | 15.9 (10.4) | 0.070 |
| Time from Hospital Arrival to Surgery, mean hour (SD) | 1.6 (0.9) | 1.4 (0.6) | 0.37 |
| Time from Hospital Arrival to Blood Transfusion, mean hour (SD) | 0.9 (0.2) | 0.8 (0.5) | 0.74 |
